# Factors associated with conducting planning for secondary oral health care services in Brazil

**DOI:** 10.1186/s12913-020-05703-7

**Published:** 2020-09-11

**Authors:** Edson Hilan Gomes de Lucena, Rênnis Oliveira da Silva, Carolina Dantas Rocha Xavier de Lucena, Amalia Issufo Mepatia, Yuri Wanderley Cavalcanti, Paulo Savio Angeiras de Goes, Maria Fátima de Sousa

**Affiliations:** 1 Federal University of Paraiba, João Pessoa, Brazil; 2 State Secretariat of Health of Paraiba, João Pessoa, Brazil; 3 Technical Advisor of the National Coordination of Oral Health, Ministry of Health, Maputo, Mozambique; 4 Department of General and Preventive Dentistry, Federal University of Pernambuco, Recife, Brazil; 5 Departament of Public Health, Faculty of Health Sciences, Center for Studies in Public Health, University de Brasília, Brasília, Brazil

**Keywords:** Oral Health, Secondary care, Health planning

## Abstract

**Background:**

Planning in health services specifically aims to improve the health status of a given population, guaranteeing access with equity and justice, as well as streamlining the response of the health system to the needs perceived by the community. This research aims to identify the factors associated with planning Specialized Dental Clinics (SDCs).

**Methods:**

Secondary data were used from the external evaluation of the database of the first National Program for Access and Quality Improvement of SDCs (NPAQI–SDCs) and the informed Outpatient Information System of the Unified Health System (OIS/UHS), which contains data on the specialized dental procedures performed at SDCs. It consisted of a quantitative study in which Pearson chi-square statistical tests (p < 0.05) and a multivariate logistic regression were applied with odds ratio (OR) estimate.

**Results:**

The results indicated that the realization of planning in SDCs was associated with lower coverage of the Oral Health Team of the Familiy Health Strategy in a municipality (OR = 1.4; 95% CI: 1.0–1.9, *p* = 0.049), additional training for managers (*p* = 0.038), the practice of self-assessment (OR = 8.2; 95% CI: 5.8–11.6; *p* = 0.000) and meeting service production targets (OR = 1.9; 95% CI: 1.2–3.2; *p* = 0.011).

**Conclusion:**

The results indicate that the work processes of the SDCs, especially with regard to service management, are essential to the proper functioning of the service and the practice of planning is linked to the technical capacity and commitment of service managers.

## Background

The Smiling Brazil, launched in 2004, is a National Oral Health Policy (NOHP) that proposed to reorganize the oral health care model and expand access to oral health services and actions through different levels of care, resulting in new expectations for oral health care in Brazil [1].

Among the structure of the axes of the National Oral Health Policy, there is the Expansion and Qualification of Specialized Care through the creation and implementation of medium complexity services in oral health, the Specialized Dental Centers (SDCs) and the Regional Dental Prosthesis Laboratories (RDPLs) [2].

Starting in 2004, the public services that have received specialized attention are the SDCs. They constitute the main specialized dental care strategy in the Brazilian public system, and through them the number of specialists such as those in endodontics and periodontics that prevent dental losses has increased [3].

The offer and availability of dental services does not necessarily mean the reduction of oral health needs and inequalities among the population. This understanding is necessary for the more equitable planning of health service management [3].

Planning is a management tool used to make decisions, to direct the provision of health services and to organize actions in a logical and rational way in order to guarantee the best results and achieve the goals of a society with the minimum costs and in the shortest possible time [4].

To improve the health status of the population, health services planning should guarantee equitable and fair access, as well as allow the health system to promptly respond to the perceived needs of the community [5]. One of the strengths of planning lies in its use as a change agent [6].

Health services planning specifically aims to improve the health status of a certain population, ensure equitable and fair access, and accelerate the response of the health system to the perceived needs of the community. This will be achieved through the provision of efficient and effective health services, taking into account the resources available and the means and methods of health care available [5].

The lack of planning in health services leads them to perform their activities via inertia. They function in a disjointed way, guided only by the notion of their roles, according to the world view of each manager and his understanding of the guidelines established by the state sector policy. Consequently, the health system begins to function subjectively, fragmented and disorderly because of the many visions and operating methods that exist in it. The lack of a common and objective understanding of the intended destination causes each service professional to conduct and carry out their activities in their own way. Due to the various paths and directions, the advances of some are neutralized by the setbacks of others. In this way, the gains in quality will be negligible and the resources will surely be used inefficiently [7].

Planning is a strategic management tool that aims to qualify the care offered to the population and its considerable contribution to the organization’s reflection on a complex work process [8, 9]. The present study aims to identify the factors associated with the planning of Specialized Dental Centers (SDCs).

## Methods

The present study is of a quantitative, descriptive and analytical nature. It used the database of the first cycle of the National Program for Access and Quality Improvement of SDCs (NPAQI-SDCs), including Module II – Interview with the SDC manager and with SDC Dentists and document verification; and secondary data on the specialized dental procedures performed by the SDCs, as reported in the Outpatient Information System of the Unified Health System (OIS/UHS), with dates ranging from July to September 2013. This was the same period used by the NPAQI-SDCs to evaluate SDCs. The data are available in http://aps.saude.gov.br/ape/pmaq/ciclo1ceo/.

The SDCs are public health establishments, which are classified as Specialized Clinics or Specialized Ambulatory centers, and at the minimum, they should include the following clinical areas: oral diagnosis, with an emphasis on the diagnosis and detection of oral cancer; specialized periodontics; minor oral surgery on soft and hard tissues; endodontics and the treatment of patients with special needs. There are other procedures that may be available at these centers such as prosthetic rehabilitation, orthodontic treatments and dental implants in accordance with the population’s care demands [10]. They can be classified as Type I when they have three dental chairs, Type II when they have from four to six dental chairs, and Type III when they have seven or more dental chairs. Regardless of the Type, all SDCs should offer at least five of the specialties previously mentioned [11].

The NPAQI-SDC data were collected in loco in the first semester of 2014 during the Program’s External Evaluation stage. All SDCs qualified in that period were evaluated. We excluded 54 (5%) SDCs that were closed, under rehabilitation work, were disabled by the ministry of Health or that declined to participate in the evaluation. This resulted in a total of 930 services.

The following questions of the External Evaluation Instrument were selected: has there been any SDC planning activity in the last 12 months, has a self-assessment process been conducted by the SDC team in the last 6 months, is there a manager at the center, how long has the manager served in the SDC, and has or is the manager undergoing further training. All of these issues are specific to the following subdimensions: ‘Information about the interview (SDC manager)’, ‘Training and qualification of SDCs’ and ‘Planning and management actions for organization of the SDCs work process’.

The production data collection was done directly from the database of the Department of Information Technology of the UHS (DATASUS) [12], including information from the SIA/SUS, according to the National Health Establishment Register’s (NHER) number of authorized SDCs and program participants.

DATASUS’s TabWin program was used to tabulate the production data and then export it to the Excel program, version 2000 (Microsoft Corp.). The data were consolidated and grouped into four the specialties of Endodontics, Periodontics, Oral Surgery and Treatment of Patients with Special Needs, in accordance with the Ministry of Health Ordinance Nr 1.464/2011 [13].

An analysis of the average production of the three evaluated months was performed per group of dental procedures according to the performance targets established for each specialty and by type of service, that is, it could vary from 0 to 4 goals for each SDC. Goals are considered to be achieved for those specialties that ac hieve a normalized quantity for each specialized dental procedure group during the analyzed period.

The monthly targets per specialty for each type of Specialized Dental Center are the following. For SDC Type I, there should be 60 periodontic procedures, 7 of which should be permanent endodontic o bturation and/or the retreatment of permanent teeth with 3 or more roots; and 80 minor oral surgery procedures. For SDC Type II, there should be 90 periodontic procedures, of which 12 are related endodontic obturation and/or the retreatment of permanent teeth with 3 or more roots; 60 endodontic procedures and treatments; and 90 minor oral surgery procedures. For SDC Type III, there should be 150 periodontic procedures, 19 of which are related to endodontic obturation and/or retreatment of a permanent tooth with 3 or more roots; and 170 minor oral surgery procedures. The targets of the Treatment of Patients with Special Needs specialty were not evaluated due to problems in the registration of the procedures, in the Individualized Ambulatory Production Bulletin (I-APB), and in the data on SDCs in the Outpatient Information System (OIS/UHS) [13].

The contextual variable of the municipality was represented by the estimate of the population coverage of the Oral Health Team (OHT) of the Family Health Strategy (FHS) for 2013, the data of which is available in the Family Health Coverage History published by the Department of Basic Health Care Ministry of Health.

The data analysis occurred in two stages: descriptive and analytical. The Statistical Package for the Social Sciences (SPSS) version 13.0 (SPSS Inc., Chicago, United States) was used for this purpose. In the descriptive phase, the frequency distributions of the quantitative variables were determined. In the analytical phase, the associations were initially tested using the Pearson chi-square. Next, multivariate analysis was performed to allow for adjustments to the confounding effects. The variables that were found to be statistically significant in the first step were used in logistic regression analysis using the ENTER method.

The model evaluated in this study took the variable “perform validated planning activity” as the reference, which was verified through documents. Then, they were selected and grouped into three blocks of predisposition variables related to the context of the municipality (oral health coverage according to the Family health strategy), the service (having a manager, how long the manager has been a part of the service, complementary training of the manager and self-assessment), and meeting goals (at least 3 of the 4 goals required for SDCs).

Given the magnitude of the percentage of the response variable and the different levels that independent variables represent, it should be noted that the odds ratio (OR) measures produced by this technique may overestimate the associations. For this, adjusted and unadjusted measures were calculated; and for all analyses, 5% was considered significant.

The external Evaluation of the NPAQI-SDC was conducted in accordance with the standards required by the Declaration of Helsinki and were approved by the Research Ethics Board of the Health Sciences Center of the Federal University of Pernambuco under registration number 740.974 e CAAE 23458213.0.0000.5208.

## Results

Planning was observed in 77.7% of the surveyed services. This percentage varied from the regional point of view with the highest percentages in the Central-West Region (88.7%) and South (82.1%) and the lowest in the North Region (67.8%).

It was observed that 59.2% (551) of the SDCs are located in municipalities with oral health team coverage according the Family Health Strategy (OHT/FHS), which cover less than 63% of the population. It was also verified that 33.3% (310) of the SDCs have managers with complete training in the public health management area and 73% (680) demonstrated the self-assessment activity. Regarding achieving the established goals for each specialty, 26.7% of the SDCs achieved at least 3 out of the 4 evaluated goals (Table 1).

**Table 1:** Frequencies, percentages and *p*-values of independent variables in relation to planning the service in Brazil

Independent Variables	Planning realization				Total		*P*-Value
	No		Yes				
	N.	%	N.	%	N.	%	
**Proportion of Population Coverage OHT/FHS**							0.037*
< 63%	111	20.1	440	79.9	551	100	
> 63%	96	25.3	283	74.7	379	100	
Total	207	22.3	723	77.7	930	100	
**Complementary training**							0.000*
Not applicable	42	36.8	72	63.2	114	100	
No	58	27.0	157	73.0	215	100	
Yes, in public management	22	16.1	115	83.9	137	100	
Yes, collective health	28	16.2	145	83.8	173	100	
Yes, others	57	19.6	234	80.4	291	100	
Total	207	22.3	723	77.7	930	100	
**Self-assessment**							0.000*
No	123	49.2	127	50.8	250	100	
Yes	84	12.4	596	87.6	680	100	
Total	207	22.3	723	77.7	930	100	
**Achievement of goals**							0.000*
0	75	27.7	196	72.3	271	100	
1	60	26.7	165	73.3	225	100	
2	44	23.7	142	76.3	186	100	
3	13	9.1	130	90.9	143	100	
4	15	14.3	90	85.7	105	100	
Total	207	22.3	723	77.7	930	100	

*Pearson’s chi-square Test

*N.* number

Significant differences (*p* < 0,05) were found when analyzing the variables related to the municipality, the characteristics of the service and the fulfillment of the goals with the accomplishment of some planning activity in the last 12 months. Therefore, for municipalities with OHT/FHS coverage lower than 63%, services that have managers with complementary training in public management and who carried out self-assessment activities tend to plan actions. Furthermore, they achieved more goals associated with conducting planning (Table 1).

The results of the multivariate analysis performed through the logistic regression showed that planning in the SDCs is strongly associated with lower OHS/FHS coverage (<63%) (OR = 1.4; 95% CI:1.0–1.9; *p* = 0.049), the complementary training of the SDC manager (*p* = 0.038), self-assessment (OR = 8.2; 95% CI:5.8–11.6; *p* = 0.000) and the achievement of three goals (OR = 1.9; 95% CI:1.2–3.2; *p* = 0.011).

Within the proposed model, the characteristics of the service, having a manager and the time serving in the management of the SDC were not statistically significant. The only variable related to the service manager that was statistically significant was complementary training. However, when analyzing the categories of this variable individually, it can be seen that it was not significant; however, when analyzed in a global way, it is statistically significant (p = 0.038) in relation to the planning. For this reason, it was included in the explanation of the proposed model.

The manager’s time in the SDC was statistically significant in the unadjusted model, mainly for those services where the manager had served for 3 to 4 years in the role. However, this effect decreased and lost its significance (*p* = 0.066) after the result was adjusted for the municipality context, the characteristics of the service and the achievement of goals.

Similar to the previously mentioned variable, the “existence manager in the SDC” was statistically significant in the model only when it was not adjusted. Due to this situation, when analyzing the results of this variable, there is an indication that not having a manager prevents planning since the category “there is no manager” presented an OR less than 1 (*p* = 0.5), thus generating a protective effect on the variable. After adjusting the results according to the characteristics of the service and the achievement of goals, this variable loses its significance (Table 2).

**Table 2:** Multiple logistic regression of the planning performance in Dental Specialty Centers in Brazil

Variables	Not Adjusted		Adjusted by the municipality context		Adjusted by service characteristics		Adjusted by the achievement of goals	
	OR (95% CI)	***p***-Value	OR (95% CI)	***p***-Value	OR (95% CI)	***p***-Value	OR (95% CI)	***p***-Value
**Estimated Population Coverage Ratio OHT/FHS**								
< 63%	1.3 (1.0–1.8)	0.027*	1.3 (1.0–1.8)	0.027*	1.4 (1.0–1.9)	0.024*	1.4 (1.0–1.9)	0.049*
> 63%	1		1		1		1	
**Existence of manager in the SDC**								
Yes, professional who is exclusively present as the manager of the SDC	1				1		1	
Yes, professional who conducts clinical activities at the SDC	1.0 (0.7–1.3)	0.857			1.1 (0.8–1.6)	0.442	1.1 (0.8–1.6)	0.442
There is no manager	0.5 (0.3–0.7)	0.000*			0.7 (0.3–1.6)	0.394	0.7 (0.3–1.6)	0.394
**Time that the manager has served at the SDC**								
Less than a year	1				1		1	
1 to 2 years	1.5 (1.0–2.1)	0.031*			1.5 (1.0–2.1)	0.058	1.4 (1.0–2.1)	0.070
3 to 4 years	1.7 (1.0–2.9)	0.044*			1.4 (0.8–2.6)	0.223	1.4 (0.8–2.5)	0.293
5 to 9 years	1.0 (0.7–1.6)	0.897			1.0 (0.6–1.7)	0.976	1.0 (0.6–1.7)	0.967
10 years or more	5.1 (0.6–42.1)	0.130			7.8 (0.7–86.1)	0.094	7.6 (0.7–85.3)	0.100
Does not know/Not applicable	0.7 (0.4–1.0)	0.041*			1.0 (0.5–2.0)	0.934	0.8 (0.5–1.3)	0.323
**Complementary manager training**								
Yes, in collective health	1				1		1	
Yes, in public management	1.1 (0.7–1.9)	0.632			1.3 (0.7–2.3)	0.346	1.4 (0.8–2.4)	0.223
Yes, others	0.7 (0.5–1.0)	0.059			0.8 (0.5–1.2)	0.316	0.9 (0.6–1.4)	0.637
No	0.4 (0.3–0.7)	0.000*			0.6 (0.4–1.0)	0.030*	0.7 (0.4–1.0)	0.061
**Self-assessment**								
Yes	8.7 (6.2–12.2)	0.000*			8.2 (5.8–11.5)	0.000*	8.2 (5.8–11.6)	0.000*
No	1				1		1	
**Achievement of goals**								
No goals	1						1	
1 goal	1.3 (0.9–1.9)	0.107					1.1 (0.7–1.6)	0.727
2 goals	1.2 (0.9–1.8)	0.264					0.9 (0.6–1.3)	0.540
3 goals	2.6 (1.6–4.0)	0.000*					1.9 (1.2–3.2)	0.011*
4 goals	1.9 (1.2–3.0)	0.010*					1.3 (0.8–2.3)	0.297

* *p* < 0,05

## Discussion

It is essential to develop work processes that enable the creation and management of an oral health care network [14]. Otherwise, a fragmented health care model will persist in which the services of the system are not articulated, the centers act as isolated points and, consequently, they do not continuously and longitudinally respond to the demands of the population [15, 16].

In this sense, it is important of planning in health services since it establishes coordination and integration among the different levels of oral health care in order to detect, analyze and seek systematized process solutions and improvements mainly for unstructured and fragmented processes [17].

The multivariate analysis of the confounding factors, which was made possible by the use of a logistic regression, allowed us to demonstrate that the execution of planning in an SDC is associated with lower OHT/FHS coverage in the municipalities. This indicates that the performance of this activity is more linked to the behavior and technical capacity of service managers than the existence of a service network.

When analyzing the distribution of an OHT according to the population size of Brazilian municipalities, the largest share of the Family Health teams are in municipalities of up to 30 thousand inhabitants (57% in 2002 and 50,5% in 2011) and the highest mean population coverage of the OHS (73%) is also found in this group of municipalities [18]. Thus, the smallest coverage is in the large cities. Therefore, the SDCs who carried out planning are located in the largest municipalities.

The same occurred when researched the use of planning and self-assessment in the work processes of the Family Health teams in Primary Health Care [19]. They verified that, in all regions, the frequency of responses regarding the topic of planning was lower in the municipalities with up to 50.000 inhabitants. In those municipalities with more than 100.000 inhabitants, the frequency was reversed, becoming larger.

The fact that the conformation of the oral health network does not induce planning is a negative factor since it is a management tool of extreme relevance for the assistance and organization of services in the search for quality services that positively respond to the population’s needs [20].

The absence of planning in services causes them to function in a disjointed manner; consequently, the health system starts to function in a subjective, fragmented and disordered way [7]. Public policy is not the same as operating within informality or any subjectivity [17].

Regarding the factors related to the service characteristics, the existence of the association between “complementary training of the manager” and accomplishing planning is in accordance with other studies, which suggests that health organizations have competent managers to face the challenges generated by the complexity of the health sector and the demands for quality in the services provided to the population. To perform this function, it is necessary to have the technical competence and capacity to know the plan, intervention strategies, and the programming; and understand the contract forms, the work process management, the organization of the health network and the issues related to the building infrastructure and maintenance of health facilities [21].

Still in the field of service characteristics, the strong association (OR = 8,2) found in this study model between the practice of self-assessment and service planning is in accordance with other study [20]. They point out that systemic planning is linked to other important concepts that at first it does not seem to be part of but that stand out after an evaluation of their quality considering their means and results.

It is also in line with the guidelines of Ministry of Health, which considers self-assessment as a crucial point for the development of the National Program for Improving the Access and Quality of Dental Specialties, since the processes oriented for quality improvement begin with the identification and recognition of the positive and problematic dimensions of the management work and health care teams [22].

In this perspective, that an alternative to find solutions that seek to overcome organizational, geographical and socioeconomic access barriers is to carry out an evaluation to support planning and decision-making [23].

The planning process should start with the identification of problems, that is, by conducting a self-assessment; and from that diagnosis, the intervention strategies to achieve the necessary changes should be defined [24]. In other words, when services are planned in an intuitive way without self-criticism or little systematization, they create obstacles in the socialization of the elaborated projects and, consequently, compromise the scope of the necessary [21].

By recognizing the intrinsic connections between planning and evaluating or, more specifically, self-assessment, and the importance of such connections as guiding the work process, we seek to induce dynamism and break with the tradition of planning and evaluation as fragmented and bureaucratic [25].

Another factor associated with planning in the present study was the achievement of goals, especially with respect to achieving 3 of the 4 evaluated goals. Thus, the high compliance with the goals induces the accomplishment of the planning in the service. No studies were found that used the same cut-off, a goal attainment indicator for planning. However, equivalent to this question, the low rate of utilization of SDCs is related, among other factors, to the lack of a management system with a clear definition of the targets for the provision of procedures by specialty [3].

Planning, execution and monitoring are crucial factors in the management of health services. Because they directly affect the health-disease costs, low effectiveness can greatly burden health spending, especially as human lifespans increase [17]. Therefore, without there being rationality in the effective control of the results, we will hardly have a sustainable health system and probably will not achieve desirable results in the future.

The SDCs, as the main strategy of the National Oral Health Policy (Smiling Brazil) to guarantee secondary care in Brazil, should organize themselves in health services that act as a reference for Primary Care and integrate into the local and regional planning processes [26].

Nevertheless, there are limitations inherent to research using secondary data on the production of health services, and the fact that the analysis of Secondary data from specialized procedures performed by the SDCs and data from the first cycle of the NPAQI-SDCs occurs at different times. Even so, it is worth highlighting the importance of public availability of these data and their use by researchers, health professionals and managers, which enables the process of planning and programming health actions and services. Another aspect to be considered in the positive evaluation of the manuscript is that the data were collected from robust and official bases.

## Conclusions

Using the proposed study model, it was observed that factors related to the context of the municipality, that is, coverage of the Oral Health Team of the Familiy Health Strategy; the characteristics of the service, that is, the training of the service manager and the act of self-assessment; and the accomplishment of production goals are associated with the realization of SDC planning.

Therefore, the results indicate that the work processes of the SDCs, especially with regard to service management, are essential to the proper functioning of the service and the practice of planning, an inherent field of administration, is linked to the technical capacity and commitment of service managers.

## Data Availability

The data are available in: http://aps.saude.gov.br/ape/pmaq/ciclo1ceo/

## Abbreviations

NOHP: National Oral Health Policy
SDCs: Specialized Dental Centers
RDPLs: Regional Dental Prosthesis Laboratories
NPAQI-SDCs: National Program for Access and Quality Improvement of SDCs
OIS/UHS: Outpatient Information System of the Unified Health System
NHER: National Health Establishment Register’s
I-APB: Individualized Ambulatory Production Bulletin
OHT: Oral Health Team
FHS: Family Health Strategy
OR: Odds Ratio
